# Correction: Real-world experience with molnupiravir during the period of SARS-CoV-2 Omicron variant dominance

**DOI:** 10.1007/s43440-022-00419-3

**Published:** 2022-10-03

**Authors:** Robert Flisiak, Dorota Zarębska-Michaluk, Magdalena Rogalska, Justyna Anna Kryńska, Justyna Kowalska, Ewa Dutkiewicz, Krystyna Dobrowolska, Jerzy Jaroszewicz, Anna Moniuszko-Malinowska, Marta Rorat, Regina Podlasin, Olga Tronina, Piotr Rzymski

**Affiliations:** 1 Department of Infectious Diseases and Hepatology, Medical University of Białystok, 15-540, ul. Żurawia 14, Białystok, Poland; 2 Department of Infectious Diseases, Jan Kochanowski University, Kielce, Poland; 3 Provincial Hospital, Kielce, Poland; 4 Department of Adults’ Infectious Diseases, Medical University of Warsaw, Warsaw, Poland; 5 ZOZ, Busko Zdroj, Poland; 6 Jan Kochanowski University, Collegium Medicum, Kielce, Poland; 7 Department of Infectious Diseases and Hepatology, Medical University of Silesia, Katowice, Poland; 8 Department of Infectious Diseases and Neuroinfections, Medical University of Białystok, Białystok, Poland; 9 Department of Forensic Medicine, Wrocław Medical University, Wrocław, Poland; 10 IV-th Department, Hospital for Infectious Diseases, Warsaw, Poland; 11 Department of Transplantation Medicine, Nephrology, and Internal Diseases, Medical University of Warsaw, Warsaw, Poland; 12 Department of Environmental Medicine, Poznan University of Medical Sciences, Poznan, Poland

## Correction: Pharmacological Reports 10.1007/s43440-022-00408-6

In the sections of "Abstract", "Patients and Methods” and Table 1, 31 April should be corrected to 30 April.

The original article has been corrected.

